# Whole-Genome Sequences of Eight Clinical Isolates of Burkholderia pseudomallei from Melioidosis Patients in Eastern Sri Lanka

**DOI:** 10.1128/MRA.00645-19

**Published:** 2019-08-15

**Authors:** Himali S. Jayasinghearachchi, Enoka M. Corea, Shivankari Krishnananthasivam, Harindra D. Sathkumara, Vaithehi R. Francis, Thimirangi R. Abeysekere, Aruna Dharshan De Silva

**Affiliations:** a Biomedical Laboratory 02, Department of Paraclinical Sciences, Faculty of Medicine, General Sir John Kotelawala Defense University, Ratmalana, Sri Lanka; b Department of Microbiology, Faculty of Medicine, University of Colombo, Colombo, Sri Lanka; c Genetech Research Institute, Colombo, Sri Lanka; d Department of Microbiology, Faculty of Health-Care Sciences, Eastern University, Vantharumoolai, Sri Lanka; University of Delaware

## Abstract

Here, we report whole-genome sequences (WGS) of eight clinical isolates of Burkholderia pseudomallei obtained from melioidosis patients with sepsis in eastern Sri Lanka.

## ANNOUNCEMENT

Whole-genome sequencing of Burkholderia pseudomallei, the causative agent of melioidosis, provides a better understanding about the phylogeography, transmission, evolution, virulence, epidemiology, and antibiotic resistance (1) of this organism. It is now clearly established that melioidosis is endemic in Sri Lanka with a wide geographic distribution (2). Whole-genome sequences (WGS) of B. pseudomallei are available for Southeast Asian (3) and northern Australian (4) strains. However, only a few WGS data sets have been published for the Indian subcontinent (5).

Here, we report eight complete genome sequences of clinical isolates of B. pseudomallei (BPs110, BPs111, BPs112, BPs114, BPs115, BPs116, BPs122, and BPs133) from melioidosis patients with acute sepsis in eastern Sri Lanka.

Strains were isolated from blood samples collected from melioidosis patients under sterile conditions, and blood agar base (Oxoid, UK) supplemented with 5% blood was used for the isolation of the organism. Subculturing was done several times on the same medium. One well-isolated single colony was restreaked on the fresh medium, a few well-isolated single colonies were pooled, and genomic DNA was extracted using a mini-QIAamp DNA isolation kit as recommended by the manufacturer (Qiagen, Germany). Multiple real-time PCR assays (*Yersinia*-like fimbrial/Burkholderia thailandensis-like flagellum and chemotaxis region [YLF/BTFC]) were performed (6, 7). Further, real-time *lpxo* PCR was used for confirmation of presumptive B. pseudomallei (6). High-quality genomic DNA of each isolate was subjected to whole-genome sequencing from a paired end with 300 nucleotide reads (Nextera DNA library prep kit) using the MiSeq 2000 platform at Agiomix FZ LLC in the United Arab Emirates.

Raw sequence data were processed with Trimmomatic 0.36 (8) and FASTX-Toolkit 0.0.13 (http://hannonlab.cshl.edu/fastx_toolkit/) to remove Illumina adaptor sequences and low-quality bases and reads. The quality of the raw sequence data was assessed using FastQC 0.11.4 (9) and MultiQC 1.0 (10). The Burrows-Wheeler Aligner (BWA) 0.7.12-r1039 (11) and Qualimap 2.2.1 (12) were used for raw read alignments and quality control of the alignment sequencing data. SPAdes 3.10.1 (13), ABACAS 1.3.1 (14), NCBI local BLAST 2.6.0, and online RAST (15) were used for genome assembly, annotation, and validation. All tools were used with default parameters, and cleaned sequences were used for downstream analysis. The assemblies were reorganized relative to the closed B. pseudomallei K96243 genome (GenBank accession numbers NC_006350 and NC_006351). All genomes reported here have been annotated using a best-placed reference protein set, GeneMarkS-2+, and the NCBI annotation provider (NCBI Prokaryotic Genome Annotation Pipeline (https://www.ncbi.nlm.nih.gov/genome/annotation_prok/). The genomes of the B. pseudomallei isolates reported here contain two chromosomes, and the features annotated are reported in Table 1.

**TABLE 1:** Characteristics and accession numbers of genomes of Burkholderia pseudomallei isolates sequenced in this study

Strain designation	Multilocus sequence type^*a*^	Genome size (bp) (GC content [%])	No. of ncRNAs^*b*^	No. of CDSs^*c*^	YLF/BTFC PCR^*d*^	No. of pseudogenes	No. of RNA genes	No. of tRNA genes	No. of contigs	*N*_50_ (bp)	No. of raw reads	GenBank accession no.
BPs110	1152	6,962,327 (68.39)	4	6,670	BTFC	514	77	61	132	115,980	2,192,501	CP036451, CP036452
BPs111	1364	6,721,089 (68.17)	4	6,670	YLF	513	76	60	147	113,263	2,007,588	CP036453, CP036454
BPs112	1442	6,258,284 (68.36)	4	6,608	YLF	537	78	62	163	83,750	1,993,557	CP037975, CP037976
BPs114	594	6,022,338 (68.39)	4	6,638	BTFC	508	77	77	158	101,821	2,617,163	CP037973, CP037974
BPs115	1413	6,756,482 (68.24)	4	6,663	YLF	504	77	61	160	103,836	2,427,392	CP037757, CP037758
BPs116	1179	6,693,503 (68.32)	4	6,593	BTFC	512	76	61	141	106,427	2,009,396	CP037759, CP037760
BPs122	594	6,242,888 (68.36)	4	6,709	BTFC	504	77	61	129	122,561	4,042,684	CP038194, CP038195
BPs133	594	6,106,529 (68.39)	4	6,647	BTFC	509	77	61	138	122,504	3,362,629	CP037971, CP037972

a Based on the scheme at http://pubmlst.org/bpseudomallei.

b ncRNAs, noncoding RNAs.

c CDSs, protein-coding sequences.

d YLF, *Yersinia*-like fimbrial region; BTFC, *Burkholderia thailandensis*-like flagellum chemotaxis region.

### Data availability.

All of the whole-genome sequencing projects have been deposited in GenBank, and the accession numbers are given in Table 1. The raw data are also publicly accessible under the accession numbers SRR8658974, SRR8618097, SRR8741027, SRR8759108, SRR8661621, SRR8660934, SRR8867837, and SRR8867836.
